# COVID-19—The largest isolation study in history: the value of shared learnings from spaceflight analogs

**DOI:** 10.1038/s41526-020-00122-8

**Published:** 2020-10-22

**Authors:** Alexander Choukér, Alexander C. Stahn

**Affiliations:** 1 Laboratory of Translational Research “Stress and Immunity”, Department of Anesthesiology, Hospital of the Ludwig-Maximilians-University, Marchioninistrasse 15, 81377 Munich, Germany; 2 Perelman School of Medicine at the University of Pennsylvania, Department of Psychiatry, Research Section for Behavioral Regulation and Health, 1016 Blockley Hall, 423 Guardian Drive, Philadelphia, PA 19004 USA

**Keywords:** Epidemiology, Translational research, Risk factors

## Abstract

The world is currently experiencing the largest isolation experiment in history. In an attempt to slow down the spread of the COVID-19 pandemic numerous countries across the world have been shutting down economies, education, and public life. Governments have mandated strict regulations of quarantine and social distancing in an unprecedented manner. The effects of these measures on brain, behavior, neuro-humoral and immunological responses in humans are largely unknown. Life science research for space exploration has a long history in using high-fidelity spaceflight analogs to better understand the effect of prolonged isolation and confinement on genes, molecules, cells, neural circuits, and physiological systems to behavior. We here propose to leverage the extensive experience and data from these studies and build a bridge between spaceflight research and clinical settings to foster transdisciplinary approaches to characterize the neurobehavioral effects on the immune system and vice versa. These approaches are expected to develop innovative and efficient health screening tools, diagnostic systems, and treatments to mitigate health risks associated with isolation and confinement on Earth and during future exploratory spaceflight missions.

## Introduction

Throughout history infectious diseases have been one of the greatest threats to global public health. Viruses have killed by far more humans than other disease, war or natural disaster. The outbreak of the novel Coronavirus SARS-CoV-2 shows that the risk of infectious diseases can rapidly put our health systems to the test and turn the world economy, education, and public life upside down. Research is just beginning to understand the full impact of the disease. There is increasing evidence that the current pandemic affects global health and well-being in many more ways than a respiratory disease, including the central nervous system and neurological diseases^1^. The brain’s susceptibility to SARS-CoV-2 could also be a potential risk factor for Alzheimer’s disease^2^. In addition to the clinical manifestations associated with COVID-19, physical distancing policies to control the spread of the diseases have led to large-scale and unprecedented social isolation across the globe. The prevalence of social isolation and loneliness in response to the COVID-19 pandemic is currently unclear, with estimates varying between 16 and 25%^3^. It is considered the greatest international biopsychosocial emergency the world has faced for a century^4^. Recent research reviewed the psychological implications of prolonged isolation and confinement associated with quarantine, reporting posttraumatic and acute stress symptoms, confusion, and anger in response to quarantaine^5^. The interventions to mitigate the negative effects on mental health and well-being include befriending schemes, individual and group therapies, various shared activity programs, social prescription by healthcare providers, and diverse strategies using information and communication technologies, but their effectiveness and relevance to different age groups remains to be determined^3^.

Reduced sensory stimulation and sensory monotony experienced in isolated, confined, and extreme (ICE) environments are also expected to be a major risk during future spaceflight exploratory class missions^6^. The “absence of environmental stimulation” is expected to account for sleep disruptions, impaired cognitive performance, negative affect, and interpersonal tension and conflict during exploratory spaceflight missions^7^. Space agencies and their human research programs have a long history of seeking to understand the effects of isolation and confinement on astronaut health and performance. Consequently, developing strategies to mitigate the risks of adverse behavioral conditions and psychiatric disorders associated with human spaceflight are of critical importance^8^. It may therefore not be surprising that the value of shared learnings from spaceflight during COVID-19 has been emphasized in numerous news reports and social media channels, providing anecdotal evidence for the parallelism of isolation and confinement in extreme environments and in the current pandemic^9–11^. Here, we summarize the opportunities of spaceflight analogs to accelerate (1) the understanding of the neurobehavioral and immunological consequences of social isolation during the COVID-19 pandemic, and (2) the development of innovative and efficient treatment strategies to mitigate adverse behavioral conditions.

## Isolation and confinement—one of the most serious, but also least understood risks of spaceflight

More than 50 years of research in space has revealed how the unique physiological conditions and stresses of space affect almost every biological system in all kingdoms of life; from plants, to bacteria, to humans. Man evolved in a world where Newton’s apple never fails to fall and at no stage did evolution prepare us for the eventuality that we may one day be exposed to life in zero gravity while confined in a hazardous environment. Spaceflight affects the entire human body. The lack of gravity results in considerable bone loss much akin to osteoporosis and muscle wasting^12^, cardiovascular deconditioning^13^, renal stones and kidney dysfunction^14^. However, besides these physiological effects of microgravity and multiple environmental toxicants in space, the psychological stressors are exhaustive and additive: life in a tin can, lack of privacy, separation from friends and family, high workload, operational and interpersonal distress, dietary restrictions, noise, circadian disorders and sleep loss^15^. The development of adverse behavioral conditions and psychiatric disorders during future long-duration space missions (LDSM) are considered one of the most serious, but also least understood risks^6^. Isolation and confinement are significant contributors to these unmitigated risks, and can lead to mental health symptoms, impulse control deficits, workplace errors, and even increased mortality^16^.

## History of isolation experiments

The beginning of the study of isolation and confinement can be marked by Polar expeditions, providing anecdotal evidence of the psychological and physiological challenges associated with prolonged isolation and confinement such as sleep disorders, mood disturbances, depression, anxiety, paranoia, and suicide^7^. The European Space Agency (ESA) early recognized the need to study the effects of isolation and confinement on mental well-being and performance to enable safe and successful spaceflight. Since the 1990s the European Space Agency (ESA) has been consistently sponsoring studies in isolated, controlled and confined analogs (ICC), starting with the 30-day campaign “Isolation Study for the European Manned Space Infrastructure” (ISEMSI) campaign in Bergen Norway in 1990^17^, followed by the 60-day study “Experimental Campaign for the European Manned Space Infrastructure (EXEMSI) carried out at the German Aerospace Center (DLR) in Cologne, Germany in 1992^18^. In 1994 ESA cooperated with the Institute of Biomedical Problems (IBMP) in Moscow, Russia, to further the understanding of the psychophysiological effects of isolation and confinement and extended the duration of the isolation period to 135 days in the “Human Behaviour in Extended Spaceflight” (HUBES)^19^ experiment, and up to 240 days (two crews were isolated for 110 days, and one crew was isolated for 240 days, respectively) in the “Simulation of the Flight of the International Crew on Space Station” (SFINCSS) experiment. These studies lay the groundwork for the longest isolation experiment in history, i.e., the Mars500 study, which exposed six international crewmembers to 520 days of isolation and confinement in IBMP’s NEK facility in 2011^20^. The success of the isolation studies at IBMP is now continued in close collaboration with NASA and ESA in a series of isolation studies as part of the “Scientific International Research in Unique Terrestrial Station” (SIRIUS) project^21^. In 2001 NASA started to simulate operations and test spacewalk techniques using a highly unique underwater habitat (NASA Extreme Environment Mission Operations, NEEMO) with mission durations of up to three weeks^22^. Since 2013 NASA has also been supporting prolonged isolation studies using an analog habitat HI-SEAS (Hawai’i Space Exploration Analog and Simulation) on the island of Hawaii^23^. More recently, NASA has been using its Human Exploration Research Analog (HERA) facility at Johnson Space Center to investigate the effects of isolation and confinement for up to 45 days^24^. Except for several Russian studies using the NEK complex^19–21^, and Chinese experiments in Chinese CELSS (Controlled Ecological Life Support System)^25^, laboratory-based isolation studies in isolated, controlled and confined analogs (ICC) have often been rather short in duration (<60 days). Submariners and Antarctic stations can provide excellent opportunities to study the effects of prolonged isolation and confinement in real extreme environments (isolated, confined and extreme environments, ICE). Despite more than 80 Antarctic research stations, only very few qualify as high-fidelity analogs for space research. Suitable stations are characterized by a small crew sizes (i.e., <10), extended mission duration of one year or longer, and include complex logistical operations with only limited or no rescue capabilities during the Antarctic winter.

The complications associated with isolations and confinement depend on a plethora of factors including individual issues such as personality, coping strategies, psychological support, crew dynamics, and mission duration. Physical isolation may also not necessarily have any adverse effects per se. Physical isolation and perceived social isolation, the subjective distressed feeling of being alone or separated can be related, but physical isolation is not a sufficient condition for loneliness^26^. A common denominator of perceived social isolation is that it translates to physiological stress responses via the sympathetic nervous system and the hypothalamic–pituitary–adrenal (HPA) axis. Loneliness has been shown to increase chronic sympathetic tone, oxidative stress, and HPA activation, and to decrease anti-inflammatory responses, and the expression of genes regulating glucocorticoid responses, ultimately leading to glucocorticoid resistance^27^.

## Vulnerability of the brain and the immune system during isolation and confinement

Two organ systems that are profoundly challenged by stress responses pertain to our host defense and our central control: the brain and the immune system. Stress can impact nearly any brain region, but one brain structure that is of particular importance is the hippocampus. The hippocampus plays a pivotal role in episodic memory formation, mapping spatial relationships and performing navigational tasks. Given the importance of visuospatial abilities during operations such as docking, landing, exploring and navigating in new environments and on planets with low gravity, it is imperative to understand the impact of spaceflight on spatial cognition and its neural basis. Because of the high density of corticosteroid receptors in the hippocampus it is not surprising that this brain region is very vulnerable to increased stress levels^28^. Both short- and long-term social isolation has been shown to reduce hippocampal long-term potentiation in rodents^29,30^. The dentate gyrus (DG) of the hippocampus is likely to play a key role in defining the impact of stress on hippocampal functioning. We recently published data on neuroendocrine and brain changes in response to Antarctic expeditions at the German Neumayer III station characterized by multiple stressors including environmental deprivation and prolonged physical and social isolation^31^. Our data demonstrated considerable decreases in dentate gyrus volume that were associated with changes in key neurotrophins such as brain-derived neurotrophic factor (BDNF). The reductions in dentate gyrus volume were also associated with lower cognitive performance in tests of spatial processing and the resolution of response conflicts test but there was no reduction in performance in other cognitive tests relying primarily on motor speed and attention, and manual dexterity. These data confirm the impact of environmental and social variation on hippocampal plasticity in humans^32^. The distinct changes of the dentate gyrus to environmental deprivation in comparison to other hippocampal subfields is similar to findings from animal models, suggesting a possible link between hippocampal neurogenesis, stress-induced behavioral changes, and environmental deprivation^33–35^. Furthermore, whole-brain analysis using voxel-based morphometry revealed significant decreases in gray matter volume of the right dorsolateral prefrontal cortex (DLPFC), and left orbitofrontal cortex (OFC) after the 14-month expedition^31^. THE DLPFC and the OFC are pivotal for executive control such as response inhibition, working memory and cognitive flexibility^36^, but also the generation of and regulation of emotion^37^. Projections between the hippocampus and orbitofrontal cortex (OFC), and to some extent also the DLPFC^38^, can foster cross-structural communication, and might interact to influence behavior^39^. Data from the ICC analog NEK also suggest decreases in white matter integrity of the right temporoparietal junction (TPJ) after prolonged isolation and confinement^40^. Whereas these data need to be interpreted cautiously because of their cross-sectional nature (reductions in white matter integrity were not reported within subjects over time), and a very small sample size, the results raise interesting questions about the effects of sensory deprivation on the brain during isolation and confinement that should be considered in future ICE/ICC experiments. The right TPJ integrates multisensory information and has been suggested to play a critical role for reorienting of attention, i.e., being able to respond quickly to unexpected events in the surroundings, and social processes^41^. We attribute these effects to both sensory deprivation as well as lack of diverse social interactions associated with the prolonged isolation and confinement. Together, these data suggest that prolonged isolation and confinement can have considerable, differential effects on brain structures involved in various complex cognitive controls including learning and memory formation, spatial navigation, self-control, planning, problem-solving, and emotional control. Notably, these data are characterized by considerable inter-individual differences in their response to social and environmental monotony. In addition, the recovery of these changes is currently unknown. The immune system could be a critical pathway in better understanding the specific neurobehavioral phenotypes. For instance, recent NASA funded research suggests that social isolation influences cytokine levels in the hippocampus^42^. The immune system is among the largest human organs and consists of more than four trillion cells. It affects every organ and influences almost every disease state from infection, to cancer, to cardiovascular disease and bone homeostasis. Most remarkable about the immune system is its adaptability to protect us rapidly and efficiently from the pathogenesis and progression of bacterial and viral infections. Using an orchestra of innate and adaptive response, it regulates a critical and delicate balance between health and disease. Any disruption of this equilibrium can lead to life threatening infections, autoimmune diseases, and cancer. Understanding this balance is vital to ensuring adequate immunity and health, which in a case of imbalance can lead to immune aging, viral reactivation, and hypersensitivities/allergies^43^.

## Spaceflight analogs and COVID-19 quarantine: parallels and research considerations

These gradual and inter-individual effects of psychological and environmental stressors are true for astronauts^44^, overwintering crews^45^ and participants voluntarily isolated and confined for the sake of research^46^, and also thereafter, when being re-exposed to everyday life again^47^. At the same this applies as much to people on Earth exposed to chronic emotional and physical stress—as it is happening right now around the world as a consequence of the COVID-19 pandemic. The parallels between social distancing and isolation and confinement and spaceflight analogs offer opportunities for shared learnings between these settings. For example, shedding of dormant herpes virus in saliva might be a very helpful surrogate marker of immune dysfunction. This could be internationally set and include many cases as clustered in different areas. Care points or pharmacies could enable data collections on a consensual basis. In addition, crowdsourcing could be supported by smart phone-based applications collecting self-reported data. An example is the EPB (European Polar Board)-ESA project ‘CHOICEe’ for the monitoring immune deficiencies after isolation^48^, and its potential applicability also in following the impact of infections with SARS-CoV-2 and the quarantine effects. A caveat of such epidemiological studies is often the lack of pre-pandemic reference data. Cross-sectional data from health controls including pre-mission data from spaceflight and spaceflight analogs can serve as a comparison for evaluating the effects of the COVID-19 pandemic. Data collected before the pandemic could be particularly valuable for establishing such a reference. For instance, the combination of self-reported data, medical records, biospecimen and neuroimaging collected during the previous 6 months prior to the COVID-19 pandemic and medical records could be considered either as a reference for between-subject comparisons or extended by follow up data collections, to foster a pre/post comparison within subjects. In particular, investigating brain morphological changes in a longitudinal manner could provide valuable information about the effects of social isolation on the brain plasticity, and verify the data we previously reported in response to prolonged isolation and confinement in extreme environments^31^. Further, future isolation and confinement studies of healthy individuals without a history of COVID-19 will remain a critical source for maximizing the synergies between spaceflight research and the clinical manifestations associated with social isolation.

### Need for countermeasures against the detrimental effects of isolation and confinement

To mitigate the adverse health effects associated with isolation and confinement, innovative countermeasures are critically needed. This need was recognized as early as the one of the first Antarctic expeditions. The crew of the Belgian expedition “Belgica” led by Adrien de Gerlache de Gomery became the first to endure an Antarctic winter after being accidentally trapped among ice packs in the Bellinghausen Sea in 1898. Frederick Cook’s reports from that expedition can perhaps be considered as one of the observations of melancholy and depression observed during prolonged isolation and confinement. To counter these symptoms Cook prescribed two actions, which may be considered as one of first exercise and light interventions to treat depression^15,49^. First, the “baking treatment” had crew members stay in front of the stove’s fire for several hours each day^50^. The radiant heat and light associated with the fire were expected to foster relaxation and restoration, and induce positive mood. Second, Cook requested the crew to take daily walks around the ship to engage in physical activity, which became known as the “mad-house promenade”^50^. About 100 years later, there is increasing scientific evidence about the role of exercise as a means of active release, enhancing brain structure and function, and improving cognitive performance^51,52^. Acute and chronic exercise has also been shown to improve innate and adaptive immunity^52–54^. Recent research highlights physical activity is an important clinical target to sustain and improve mental health during the current pandemic^55^.

In addition to exercise, target-specific countermeasures will be needed to specifically address the risks associated with perceived social isolation and sensory deprivation. These could deliver similar restorative effects like the “baking treatment” during the Belgica expedition. Virtual reality (VR) technologies are likely to play an important role in meeting these requirements^6^. They can foster a “virtual window”, i.e., exploring virtual worlds and induce a high degree of a first-person immersive experience of “being there”^56^, which has been suggested to be of central importance for eliciting restorative effects. The positive outcomes of exposure therapies in VR which include mood induction procedures^57,58^, stress reduction^59^, treatment of PTSD^60^, anxieties^61,62^ and pro-environmentalist behavior^63^ with immersive VR altogether have demonstrated the usefulness of this medium in changing our cognitive and emotional mechanisms. Currently, various projects sponsored by DLR, ESA, NASA and TRISH have been investigating the potential of VR as a countermeasure to mitigate sensory deprivation and social isolation. For instance, the NASA sponsored project “Hybrid Training - A Sensory Stimulation Countermeasure for Long Duration Space Exploration Missions” combines physical exercise with an interactive virtual environment to enhance sensory augmentation and stimulate brain plasticity during prolonged isolation and confinement^64^. The combination of VR technologies and physical exercise was also recently recognized as a promising coping strategy to promote health and wellness during COVID-19^65^. Former astronaut Jay C. Buckey and his team developed the Dartmouth PATH Program, a series of self-help tools designed by to relieve stress, improve mood, and maintain relationships during prolonged isolation and confinement associated with spaceflight. Ongoing research currently investigates whether the PATH Program also help people cope with the emotional stresses brought on by COVID-19^66,67^. Likewise, the NASA supported project ANSIBLE (A Network of Social Interactions for Bilateral Life Enhancement) delivered a multi-modal digital toolset that leverages virtual worlds to provide a space where humans and intelligent virtual agents can be companions, advisors, provided psychological support, and share experiences^68^. It is intended to virtually connect with their family, friends, and the ground crew to provide a sense of social consistency and permanence. Another digital resource to support astronauts during exploration class missions is the Crew Interactive MObile companioN (CIMON) project supported by DLR^69^. CIMON is a virtual voice-controlled interactive assistant that uses artificial intelligence to foster social interactions between humans and machines, and support astronauts during routine tasks. Such approaches could also directly translate to immunological benefits. It has become clear that the immune system is not autonomous and is not solely affected by pathogens alone. Instead how we feel, how we interact with others, the conditions in which we live all influence its function. This interaction between our social and emotional lives and the immune system take place via the central nervous system. Stress-sensitive neural systems and cells together with humoral pathways coordinate immune cell maturation, responsiveness and their interactions^43^. Recently, an international team of ESA, NASA and IBMP experts proposed a specific and personalized immune countermeasure prescription for prospective astronauts embarking on deep-space voyage^70^. The combination of different physical, medical and behavioral interventions including therapeutics, nutrient-enriched diets, regular exercise, adequate rest, and stress-relief could be expected to go beyond supporting immunologic responses by affecting other organ systems including the central nervous system. In particular, it is possible that the full synergistic potential of the proposed measures could be further enhanced by combining it with some of the behavioral strategies outlined above. Along these lines Data collected before the pandemic could be particularly valuable for establishing such a reference. For instance, the combination of self-reported data, medical records, biospecimen and neuroimaging collected during the previous 6 months prior to the COVID-19 pandemic and medical records could be considered either as a reference for between-subject comparisons or extended by follow up data collections, to foster a pre/post comparison within subjects. In particular, investigating brain morphological changes in a longitudinal manner could provide valuable information about the effects of social isolation on the brain plasticity, and verify the data we previously reported in response to prolonged isolation and confinement in extreme environments^51^. Further, future isolation and confinement studies of healthy individuals without a history of COVID-19 will remain a critical source for maximizing the synergies between spaceflight research and the clinical manifestations associated with social isolation.

## Spinning in and out: maximizing the synergies between spaceflight and terrestrial biomedical research

**Fig. 1:**
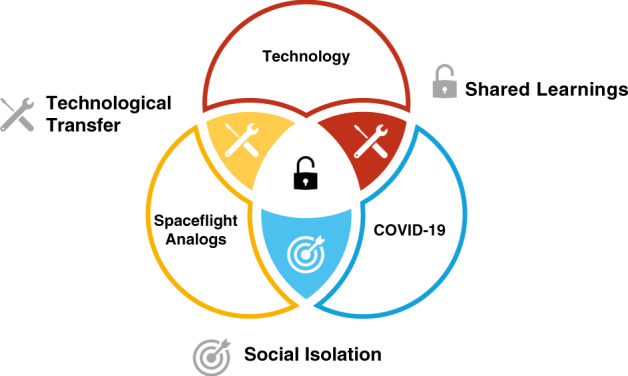
How research in spaceflight analogs and technological developments translate to the COVID-19 pandemic. Social isolation and confinement can have unique effects on brain and behavior. Physical distancing measures to reduce the spread of COVID-19 across the globe lead to an unprecedented rise in social isolation, which is considered the greatest international biopsychosocial emergency the world has faced for a century. Reduced sensory stimulation and isolation are also a major risk factor during future spaceflight exploratory class missions. Spaceflight analogs and the COVID-19 pandemic carry significant synergies for gaining a better understanding of the neurobehavioral and immunological implications of social isolation and how to mitigate related adverse health conditions. High-fidelity space analogs using Isolated, Controlled Confinement (ICC), Isolated Confinement and Extreme Environments (ICE) can foster the understanding of the effects of isolation and confinement on brain, behavior and (neuro-) immunology and assess the efficacy of treatments to mitigate adverse behavioral health conditions. This knowledge and the technologies could translate to basic research in life science and drive innovative medical and health-related applications. Vice versa, methodologies, and technologies from laboratory experiments and clinical trials can effectively transfer to improve point-of-care astronaut health and performance applications.

Spaceflight-related research offers a variety of unique experimental approaches and designs that can foster basic research in life science and drive innovative medical and health-related applications (Fig. 1). For instance, bedrest studies which can serve as a model for the aging population, and provide highly standardized and controlled conditions to assess the efficacy of new interventions and treatments to mitigate musculoskeletal, cardiovascular, and neurobehavioral risks brought by a lack of physical activity and/or immobilization^71^. Likewise, given the specific requirements of spaceflight health monitoring technologies such as size, mass, ease of operation, and non-invasiveness, human spaceflight research programs can promote health technologies on Earth. An example is the application of a heat flux-based approach to non-invasively determine core body temperature at the head^72^. The technology was validated and tested in spaceflight analogs and on ISS^73,74^. The system is now available for patient monitoring in the clinical setting because temperature monitoring is critical for treating infections, detecting complications, and reducing postoperative mortality rates^75^. At the same emerging terrestrial biomedical research and technology development can accelerate the mitigation of risks associated with human spaceflight by providing promising new approaches, treatments, countermeasures or technologies that have practical application to spaceflight.

The full scope of the synergies between terrestrial and spaceflight applications remains to be uncovered. Recent reviews highlight the need to increase the awareness, training, and collaboration of the research community for revealing the true potential of applying these space technologies to global health settings^76^. Several space agencies have begun to address this need and developed initiatives to foster productive cross-disciplinary collaboration between universities, non-university institutions, companies, and governmental and international entities. For instance, the DLR initiatives INNOspace® Masters and Space2Health seek the development of innovative space technologies and services using expertise from various industries combined with existing technologies, services and applications from space for everyday life on Earth. It promotes both the commercialization and the initiation of “spin-in” (from non-space into a space sector) and “spin-off” (from space sectors into non-space) ideas. Likewise, ESA initiated several Business Incubation Centres (ESA BICs) to inspire and work with entrepreneurs to turn space-related business concepts and ideas into commercial start-up companies. Since its start in 2003 over 700 new companies have been launched throughout Europe transfer space technologies to various applications including medical care on Earth. Another approach has been fostered by NASA’s Human Research Program (HRP) by partnering with several external entities to accelerate the risk mitigation of future long-duration spaceflight missions. This also led to the launch of the Translational Research Institute for Space Health (TRISH), a consortium spearheaded by Baylor College of Medicine and includes the California Institute of Technology in Pasadena and Massachusetts Institute of Technology in Cambridge. TRISH closely collaborates with NASA to rapidly and effectively translate knowledge, methodologies, and technologies from laboratory experiments and clinical trials to point-of-care astronaut health and performance applications. The continuation and extension of these approaches is a key factor to help bridging the gap between experts in spaceflight life sciences, basic life science, and stakeholders in health technology innovation. This is particularly true for mitigating the risks of adverse behavioral conditions associated with isolation and confinement. Along this line TRISH has announced an “Industry Program 2020” grant call for industry and academic behavioral health experts to contribute towards the development of specific key areas of interest including novel anxiety and stress monitoring techniques, and unobtrusive health monitoring technologies. Similar to the OECD Council’s “Recommendation on Responsible Innovation in Neurotechnology” the TRISH program seeks to provide guidance at each step of the innovation process so that the benefits are maximized and risks minimized. This includes providing companies and pre-companies with (1) access to experts working in space health care and at NASA, (2) rapid technology maturation and de-risking, (3) preparation for transfer to commercial healthcare markets, and (4) pathways to government sales in the emerging space market.

## Conclusions and perspectives

*“Facts are the air of scientists. Without them you can never fly”* (Linus Pauling). That is why space agencies are taking research to the skies and are supporting innovative approaches to better understand how the human body copes with extreme conditions, and how this knowledge can be transferred to and benefit the people on Earth. Here we used the parallels of spaceflight analogs and the COVID-19 pandemic to illustrate the value of shared learnings for gaining a better understanding of the neurobehavioral and immunological implications of social isolation and how to mitigate related adverse health conditions. Since space research is addressing and increasingly influencing the traditional fields of medicine including neurobiology, clinical immunology, and public health of an aging society, this pandemic, the re-entry to normal life and its aftermath should strengthen current inter- and transdisciplinary collaborative research initiatives between space life sciences, basic biomedical science, clinical applications, and industrial stakeholders to mitigate the negative effects of physical and social isolation in health and disease. Such collaborative research initiatives combined with historic data from spaceflight analogs could lead to a better understanding of the cause and effects of social distancing and quarantine on health and mental well-being. They can also provide the basis to develop innovative and efficient health screening tools, diagnostic systems, and personalized treatments to mitigate health risks associated with isolation and confinement. Partnerships between space agencies, non-space funding bodies, academic institutions and companies will continue to play an important role to translate research from Space to Earth and scale it effectively.
